# Quality of life in colon cancer patients with skin side effects: preliminary results from a monocentric cross sectional study

**DOI:** 10.1186/1477-7525-8-40

**Published:** 2010-04-15

**Authors:** Federica Andreis, Anna Rizzi, Paola Mosconi, Claudia Braun, Luigina Rota, Fausto Meriggi, Maria Mazzocchi, Alberto Zaniboni

**Affiliations:** 1 UO di Oncologia Medica, Fondazione Poliambulanza, Via Bissolati 57, 25124 Brescia, Italy; 2 Istituto di Ricerche Farmacologiche Mario Negri, Via La Masa 19, 20156 Milano, Italy

## Abstract

**Background:**

Epidermal growth factor receptor inhibitors are widely prescribed anticancer drugs. Patients treated commonly develop dermatologic adverse drugs reactions, but rarely they are involved in systematic evaluation of their quality of life. This monocentric cross sectional study is carried out to assess quality of life in colon cancer patients experienced skin side effects due to anti epidermal growth factor receptor inhibitors therapy.

**Methods:**

Consecutive patients with skin side effects to therapy treated at Fondazione Poliambulanza were enrolled in this study. Quality of life was evaluated with the Italian validated version of Skindex-29 questionnaire, exploring three dimensions: symptoms, emotional, and physical functioning. Skindex-29 was administered one time between the eighth and the twelfth week of the treatment.

**Results:**

Forty-five consecutive patients, mainly with metastatic colon cancer (29 female, 16 male), with an average age of 59.31 years (ranging from 34-78) were included in the study and analyzed. Patients showed a great impact of skin side effects on symptoms (mean 43), followed by emotional (mean 30), and functioning (mean 26) scales. In general women, the 55-65 age class, and patients with partial remission reported the worst quality of life.

**Conclusions:**

Epidermal growth factor receptor inhibitors' skin side effects have an important impact on quality of life in advanced colon cancer patients; symptoms scale is the most effect respect to emotional and functioning scales.

## Background

Epidermal growth factor receptor (EGFR) inhibitors, as cetuximab or panitumumab, have become widely prescribed anticancer drugs for the treatment of colorectal, head and neck and lung cancer, alone or in combination with traditional chemotherapy [1]. Patients treated with EGFR inhibitors commonly develop a wide range of dermatologic adverse drug reactions, including a papulopustular rash, dry skin, itching and alterations in hair and periungual tissues, which can result in a decreased quality of life (QoL) [2]. The rash can occur in 50-90% of patients treated, arising primarily on the face, and appearing similar, but non identical, to acne. The rash can be painful and the paronychial cracking, the paper-cut feeling in the fingers and toes can become very disturbing, and could interfere with the daily activities of a relevant proportion of patients [3]. Moreover many patients experience significant skin side effects and find that these are the first physical appearance of the disease; this situation could imply that many patients withdraw from social activities because of the impact on their appearance and their concerns about how others would react. As a consequence, specific skin toxicities associated with EGFR inhibitors can have a profound impact on patient's physical, emotional and social functions, the typical dimensions assessed in QoL evaluation. Some data reported in the literature regard cancer or colon cancer patients that experienced skin side effects, but rarely patients are requested to evaluate the impact of these problems on their life and activities, or to participate in a QoL survey [4,5].

The present study was carried out to evaluate the impact on QoL in a population of patients with advanced colon cancer who experience at least grade II skin side effects according with National Cancer Institute-Common Terminology Criteria (NCI-CTC). We used the Italian version of a well-known dermatological instrument, the Skindex-29 questionnaire [6-8], which is able to better describe and score the real impact of skin toxicities on daily QoL.

## Methods

This cross sectional study was conducted at the Oncological ward of the Fondazione Poliambulanza from March to December 2008. Consecutive patients, not enrolled in clinical trial, mainly with metastatic colon cancer, who experienced, during the EGFR inhibitors treatment, at least a grade II skin side effects scored as reported in Table 1, were asked to participate in the study. The study was approved from the Institutional Review Board of the hospital, and all patients were provided with a written informed consent before entering the study. Eligible patients were Italian speaking older than 18 years of age. All patients were informed on their diagnosis and prognosis, information was conveyed thought medical consultations, and written material.

**Table 1:** National Cancer Institute common terminology criteria for grading selected dermatologic adverse events*

Grade	Dry Skin	Nail changes	Pruritus/Itching	Rash/Desquamation	Rash: Acne/Acneiform
**1**	Asymptomatic	Discoloration, ridging, pitting	Mild or localized	Macular or papular eruption, or erythema without associated symptoms	Intervention not indicated

**2**	Symptomatic, not interfering with activities of daily living (AOL)	Partial or complete loss of nail(s); pain in nails	Intense or widespread	Macular or papular eruption, or erythema with pruritus or other associated symptoms; localized desquamation or other lesions covering < 50% body surface area (BSA)	Intervention indicated

**3**	Interfering with ADL	Interfering with ADL	Intense or widespread and interfering with ADL	Severe, generalized erythroderma, or macular, papular or vesicular eruption; desquamation covering > 50% BSA	Associated with pain, disfigurement, ulceration, or desquamation

**4**	-	-	-	Generalized, exfoliative, ulcerative, or bullous dermatitis	-

**5**	-	-	-	Death	Death

* From Common terminology criteria for adverse events v3.0, by the National Cancer Institute, 2003. Retrieved September 2, 2005, from http://ctep.cancer.gov/reporting/ctc.html. Reprinted with permission

Following a literature review [9], the Italian version of Skindex-29 was chosen to evaluate the impact of skin reactions considering its documented validity and reliability [6-8]. This questionnaire has been previously validated in Italian language as described in detail elsewhere [10]. Skindex-29 is a disease-specific self-administered questionnaire that measures the complex effects of skin disease on patients' QoL through three multi-item scales: physical symptoms (7 items), emotional state (10 items), and social function (12 items). Answers to each item are given on 5-point scale, from 'never' to 'all time'. For each scale the score is calculated as the mean of response to the items included in the scale and the scale scores are standardized to 100 [8]. According with the literature, when missing data were less than 20% of the items included in a scale, missing value was substituted by the mean of the total score of the scale [11]. Higher scores indicate an increase impact of skin side effects on QoL, i.e. a worse patient's QoL.

After informed consent had been obtained, the Skindex-29 questionnaire was presented by a trained interviewer (AF); patients completed the self-administered questionnaire during a routine visit but before medical examination in order to reduce possible influence on patient responses.

The questionnaire was administered between the eighth and the twelfth week of the treatment, only one time for each patients, according to a cross-sectional design.

Statistical analyses were performed by SAS^® ^System. Clinical and demographic variables were described using descriptive statistics such as mean, standard deviation and proportion. Frequency distributions of survey questions were obtained by all the sample. In addiction we constructed the three scales' index - symptoms, functioning and emotional - using the mean of the total score of the scale and standardizing scores to 100. The mean of the scales is analysed in function of sex, age (three levels), clinical outcomes, and age classified by sex.

## Results

Among ninety-four screened patients, forty-five consecutive colon cancer patients experienced at least a grade II skin toxicity and were therefore enrolled in the study. The Skindex-29 has been very well accepted by this sample of cancer patients since it hasn't been any refuse to participate, and we registered only three missing answers at item level. The estimated Cronbach coefficient alpha value for these colon cancer patients were .85, .84, .89 for symptoms, emotion, and functioning respectively.

The demographic characteristics of patients are listed in Table 2. There were 29 female (64%) and 16 male (36%) with an average age of 59.31 years (ranging from 34 to 78 years). High-school graduation and university education was achieved in the 36% of the patients enrolled, most of patients are married. Thirty of forty-five patients received the treatment as second or further lines for metastatic disease; one third of patients experienced a grade 3 skin toxicity (14 subjects), two thirds a grade 2 skin toxicity (31 subjects).

**Table 2:** Characteristics of sample considered, n° = 45

		N°	%
***Sex***	Female	29	64.4

	Male	16	35.6

***Age (year)***	Mean and standard deviation (min-max)	59.31 ± 9.94 (34-78)	

***Education***	Elementary	13	28.8

	Secondary school	16	35.6

	High school	16	35.6

***Status***	Married/live together	39	86.7

	Single	6	13.3

***Treatment***	Adjuvant	5	11.1

	First line	9	20.0

	Second line	18	40.0

	Third line	12	26.7

	Other	1	2.2

***Outcome***	Partial remission	16	35.6

	Stable disease	14	31.1

	Progressive disease	10	22.2

	Not evaluable	5	11.1

The three health related QoL scores measured with the Skindex-29 questionnaire in our population showed a great impact of skin side effects on symptoms (mean 43), followed by emotional (mean 30), and functioning (mean 26) scales, as reported in Table 3. All the three scores are higher (worst QoL) in the subgroup sample of females; the worst scores are in the 55-65 age class. When data were analysed according with sex and age, the class of women aged 55-65 years have the higher value of symptoms, emotional and functioning scales. Patients with partial response have the worst QoL. As expected patients with 3 grade toxicity reported a worst scores (worst QoL) for all the scales considered respect to patients with 2 grade toxicity, however the most relevant difference is related to the symptoms scale.

**Table 3:** Skindex-29 scores by patients characteristics

	SKINDEX-29 SCALES		
	
	Symptoms Mean SD (min-max)	Emotional Mean SD (min-max)	Functioning Mean SD (min-max)
***Total sample ****(n° = 45)*	43.33 ± 20.22 (07-96)	30.33 ± 14.16 (05-67)	26.85 ± 17.57 (0-62)

***Sex***			
*Male (n° = 29)*	41.87 ± 18.55 (07-82)	29.31 ± 12.98 (05-57)	24.71 ± 16.74 (00-56)
*Female (n° = 16)*	45.98 ± 23.26 (07-96)	32.18 ± 16.35 (05-67)	30.73 ± 18.93 (00-62)

***Age - 3 levels***			
*34-54 years (n° = 13)*	42.58 ± 20.64 (07-71)	29.80 ± 14.45 (05-50)	24.67 ± 21.97 (00-60)
*55-65 years (n° = 19)*	46.05 ± 23.20 (07-96)	32.23 ± 16.24 (05-67)	29.49 ± 15.82 (04-62)
*> 65 years (n° = 13)*	40.10 ± 15.57 (07-71)	28.07 ± 10.90 (05-50)	25.16 ± 15.96 (00-52)

***Age 3 and Male***			
*34-54 years (n° = 6)*	48.80 ± 13.67 (25-64)	34.16 ± 09.57 (17-45)	19.79 ± 18.71 (04-54)
*55-65 years (n° = 13)*	42.30 ± 23.04 (07-82)	29.61 ± 16.13 (05-57)	26.92 ± 16.12 (04-56)
*> 65 years (n° = 10)*	37.14 ± 14.20 (07-53)	26.00 ± 09.94 (05-37)	24.79 ± 17.52 (00-52)

***Age 3 and Female***			
*34-54 years (n° = 7)*	37.24 ± 24.98 (07-71)	26.07 ± 17.49 (05-50)	28.86 ± 25.09 (00-60)
*55-65 years (n° = 6)*	54.16 ± 23.41 (28-96)	37.91 ± 16.38 (20-67)	35.06 ± 14.93 (20-62)
*> 65 years (n° = 3)*	50.00 ± 18.89 (35-71)	35.00 ± 13.22 (25-50)	26.38 ± 12.02 (12-33)

***Clinical outcome***			
*Partial remission (n° = 16)*	45.98 ± 18.98 (07-71)	32.18 ± 13.28 (05-50)	28.51 ± 19.55 (00-56)
*No remission (n° = 29)*	41.87 ± 21.06 (07-96)	29.31 ± 14.74 (05-67)	25.93 ± 16.68 (00-62)

***Grade skin toxicity***			
***2 ****(n° = 31)*	39.28 ± 19.64 (07-82)	27.50 ± 13.75 (05-57)	23.11 ± 16.28 (00-60)
***3****(n° = 14)*	52.29 ± 19.18 (18-96)	36.60 ± 13.43 (12-67)	35.12 ± 18.08 (06-62)

Since the subgroups analysis showed very small sample of patients, we couldn't perform analysis to test statistical significance differences.

## Discussion

Psychological effects of anti-EGFR-induced cutaneous toxicities are increasingly recognized as an important issue to cope with for both patients and physicians [2,3]. Nevertheless, up till now there is a substantial lack of prospective clinical trials, reporting data on QoL during anti-EGFR therapy in colon cancer patients. Recently, Au et al reported the QoL of patients with advanced colorectal cancer treated with cetuximab, utilizing the EORTC QLC-C30 questionnaire. In this study, cetuximab offered an important benefit in terms of QoL compared with best supportive care alone [12]. Interestingly, Peeters et al, suggested that panitumumab monotherapy is associated with better QoL scores, using a modified Dermatology Life Quality Index questionnaire, and supported the use of dermatological-oriented questionnaire (like Skindex-29) to better capture key-symptoms potentially harmful for QoL in this setting [13]. In Table 4, a comparison with available literature data is presented where not only oncological patients are considered [8,11,14,15]. Respect to other conditions, the impact of skin side effects on colon cancer patients is relevant for all the three scales considered. In particular the impact on symptoms is as important as that recorded in patients with psoriasis, while when compared to dermatological diseases as psoriasis, eczema, or acne vulgaris the impact on the emotional scale is lower.

**Table 4:** Comparison with literature data

	SKINDEX-29 SCALES		
	
	Symptoms Mean	Emotional Mean	Functioning Mean
Metastatic colon cancer patients (45)	43	30	26

Psoriasis (45) [8]*	41	38	22
Eczema (103)	48	41	25
Benign growth (75)	21	21	8
New melanoma cancer (137)	27	20	9

Acne vulgaris (57) [14]**	29	39	15
Psoriasis (44)	42	38	24
Benign lesion (75)	23	21	8
Normative sample (107)	13	9	3

Cutaneus T-cell lymphomas (22) [11]	19	23	24

First diagnosis of cutaneus B-cell lymphomas (24)	15	20	5
First diagnosis of micosis fungoide (59)	25	28	15
First diagnosis of Sezary syndrome (12) [15]***	70	52	45

() in the brackets the number of patients considered

* data from figure six of the original article

** data from figure two of the original article

*** data from figure three of the original article

It should be underlined that there is a direct association between the development of skin toxicities (mainly rash) and the probability of getting a good response from the treatment [16,17]. Patients are generally informed about this kind of correlation since the start of the treatment and this awareness should be taken into account in the coping process of patients with their toxicities. Our study documents in a sample of advanced colon cancer patients the impact of skin side effects on symptoms, emotional and functioning scales, with the impact on symptoms worse than other scores scales. Interestingly scores vary according with sex, age, the achievement of partial remission, and grade of toxicity suggesting to reserve special attention to psychological support, doctor-patient relationship and in providing these groups of patients with accurate and truthful information. In respect to comparative data of subjects with skin clinical problems, our sample of colon cancer patients showed in general a worse impact on symptoms and functional QoL scales. Surprisingly, the impact on emotional scale is less important when compared with patients with other skin problems. The severity of clinical condition (mainly metastatic colon cancer patients), the impact of the disease and relapse may partially explain this different focus on the items considered in the emotional scale. It is reasonable to think that some items like "I worry that my skin condition may be serious (item 3), "...makes me feel depressed" (item 6), or "I am angry about my skin condition" (item 15) may affect differently in a population with a limited perspective of survival as patients in this study.

As expected, the worst scores were noted in responding and in 3 grade toxicity patients, considering the link between skin toxicity and drug activity, as mentioned before and reported by literature [16,17]. It could be that the lower than expected impact of treatment on emotional, symptom, functioning scales is due to the awareness of correctly informed patients of the relationship between intensity of toxicity and positive response to the treatment. This awareness may, to some extent, mitigate the significance of certain items like shame, social isolation, sexuality.

Currently there is no evidence-based treatment guideline to prevent or treat the EGFR inhibitor-associated skin toxicities, except for a recently reported strategy of preventive treatment considered in a small randomized study [18-20]. At the moment consensus emphasizes the importance of developing an interdisciplinary approach involving specialists in oncology and dermatology. Nevertheless, selected patients might benefit also from early psychological support.

We are aware that our study has some limitations: the sample size of this study is not very large, so the data analysis is partial. However our patients population is prospectively studied on a consecutive basis and might be a quite representative sample of real-world clinical practice outside clinical trials. Another limitation is that this study is cross-sectional: QoL has been evaluated at only one time point instead of prospectively evaluating toxicities' impact on aspects of life over time. Finally, considering the clinical conditions of patients included in this study (mainly metastatic and cancer) it was not considered feasible in our setting to administer both a specific and a generic questionnaire. For these reasons we decided to use only one QoL questionnaire - the dermatological specific instrument Skindex-29 - which is able to better describe and score the real impact of skin toxicities on daily QoL, compared to generic tools as EORTC QLQ C-30 or SF-36.

## Conclusion

This study documents the impact of skin side effects on quality of life of advanced colon cancer patients treated with EGFR inhibitors. Among the evaluated scales considered by Skindex29 questionnaire, the symptoms scale is the most effect respect to emotional and functioning ones.

Future studies should also explore the combined use of usually employed generic questionnaires and dermatologic-oriented tools, like Skindex-29, in an attempt to better define the ultimate impact of EGFR therapeutic approaches involving a constantly increasing number of cancer patients.

## Competing interests

The authors declare that they have no competing interests.

## Authors' contributions

All the authors actively participated to plan the study described in the article. In particular:

AF, RA, RL, MF, DBB, AC and MM presented the study to patients, collected and assembled data. BC and MP perform the data analysis and discussed results with other authors. ZA and MP drafted the article and all the authors reviewed it and gave final approval of the version to be published.
